# Association Between Serum Concentrations of (Certain) Metals and Type 2 Diabetes Mellitus

**DOI:** 10.3390/jcm13237443

**Published:** 2024-12-06

**Authors:** Magdalena Tyczyńska, Gabriela Hunek, Weronika Kawecka, Adam Brachet, Marta Gędek, Kinga Kulczycka, Katarzyna Czarnek, Jolanta Flieger, Jacek Baj

**Affiliations:** 1 Department of Correct, Clinical and Imaging Anatomy, Medical University of Lublin, Jaczewskiego 4, 20-090 Lublin, Poland; hunekgabriela@gmail.com (G.H.); weronika.kaw98@gmail.com (W.K.); 2 Department of Forensic Medicine, Medical University of Lublin, Jaczewskiego 8b, 20-090 Lublin, Poland; adambrachet@gmail.com (A.B.); gedekmarta@gmail.com (M.G.); 3 Institute of Medical Sciences, The John Paul II Catholic University of Lublin, Konstantynów 1, 20-708 Lublin, Poland; kinga.kulczycka@kul.pl (K.K.); katarzyna.czarnek@kul.pl (K.C.); 4 Department of Analytical Chemistry, Medical University of Lublin, Chodźki 4A, 20-093 Lublin, Poland; jolanta.flieger@umlub.pl

**Keywords:** diabetes mellitus type 2, trace elements, metals, micronutrients

## Abstract

The findings regarding trace element concentrations in patients diagnosed with type 2 diabetes and healthy controls are inconsistent, and therefore, we determined to gather them in the form of a review to further indicate the need for more advanced knowledge development. In our study, we reviewed articles and studies that involved the topics of micronutrient and metal associations with the occurrence and development of type 2 diabetes. We mainly included works regarding human-based studies, but with limited research results, animal-based research was also taken into account. With some newer studies, we reached for initial assumptions of previous statements. The results indicated that higher serum levels of lead, cadmium, arsenic, bromine, barium, strontium, nickel, aluminum, calcium, copper, and ferritin are positively associated with diabetic prevalence. Both too-low and too-high levels of zinc, selenium, and magnesium may be connected to the development of diabetes. Chromium has the capability of insulin response modulation, with enhanced insulin-cell binding, and thus, lower serum levels of chromium can be found in diabetic patients. There are contradictory discoveries regarding manganese. Its supplementation can possibly cease the development of insulin resistance and type 2 diabetes. On the contrary, other studies reported that there is no such connection. Our work indicates that, as micronutrients play a significant role in the pathogenesis of metabolic disorders, more research regarding their bodily homeostasis and type 2 diabetes should be conducted.

## 1. Introduction

Diabetes mellitus is one of the most frequent metabolic disorders, characterized by elevated levels of blood glucose. Over 90% of cases are type 2 diabetes mellitus (T2DM), whose development is primarily caused by a combination of a defective insulin secretion by pancreatic β-cells and the inability of insulin-sensitive tissues to respond to insulin [1,2,3]. The prevalence of T2DM has increased from 151 million adults worldwide in 2000 to 463 million in 2019 and is expected to approach around 693 million cases in 2045 [1,4,5,6]. The highest percentage of new incidences of T2DM is observed in countries in the Middle East, North Africa, and South Asia [5,7,8]. The incidence of T2DM was approximately 6.0% in men and 5% in women in 2019 [5]. Moreover, its occurrence increases with age, with patients between 55 and 59 years old being the most susceptible, manifesting slightly earlier in men than in women [9,10,11].

The main mechanisms leading to T2DM are a complex combination of insulin resistance (IR) and initial hyperinsulinemia, followed by progressive dysfunction of pancreatic β-cells to produce insulin [2,12,13,14]. IR is a reduced response of insulin-targeting tissues to high physiological insulin levels [15]. The three most important extra-pancreatic insulin-sensitive organs that are associated with the development of T2DM include the skeletal muscles, adipose tissue, and liver [2,12,13]. Mutations that reduce the expression of insulin receptors or GLUT4 in the skeletal muscle cells, as well as any defect disturbing the signaling pathway, would reduce glucose intake into the muscle and lead to hyperglycemia [2,16]. An impaired response to insulin by adipose tissue leads to the impaired suppression of lipolysis, impaired glucose uptake, and enhanced free fatty acids (FFA) release into the plasma, even if the insulin level is high [17]. The elevated release of FFA accumulates in other tissues, especially in the liver, which causes impaired insulin signaling, increased hepatic gluconeogenesis, and impairment of the glucose-stimulated insulin response. All of these factors induce T2DM development [2]. The dysfunction of β-cells is associated with β-cell death and is an outcome of inflammation, inflammatory stress, ER stress, metabolic/oxidative stress, and amyloid stress [14].

The complex pathophysiological mechanisms are strongly associated with multiple risk factors for T2DM development. The risk factors of T2DM include both non-modifiable (ethnicity, family history, genetic predisposition) and modifiable (obesity, low physical activity, an unhealthy diet, hypertension, dyslipidemia) factors. Evidence from epidemiological studies suggests that improving the main modifiable risk factors can prevent many cases of T2DM [13,18,19].

The most significant complications and outcomes of T2DM are higher cardiovascular risk, diabetic dyslipidemia, atherosclerosis, endothelial dysfunction, and diabetes-associated chronic inflammation [2,13]. Moreover, T2DM is associated with increased risk of both macrovascular (ischemic heart disease, cerebrovascular disease, peripheral vascular disease) and microvascular (neuropathy, nephropathy, retinopathy) complications [13]. All of these factors negatively affect quality of life [20].

Studies indicated different concentrations of trace elements between patients diagnosed with type 2 diabetes and healthy controls, but the results have not been consistent. Trace elements present different ways of affecting the pathogenesis of diabetes. Disturbance in the homeostasis of specific trace elements may impact both glucose and insulin metabolism and increase oxidative stress reactions, which may further lead to insulin resistance development. In our study, we determined to gather and review the information about already known and established associations to further indicate the need for advanced knowledge development in this field. We mainly focused on meta-analysis, profound review articles, and original studies. Human-based research was our priority, whereas in times when the data was difficult to obtain, we opted for animal-based research.

In the performed studies, chromium showed the capability of insulin response modulation through the enhancement of insulin-cell binding, an increased number of insulin receptors, and the activation of the insulin receptor kinase [21]. Zinc is involved in insulin synthesis, storage, and secretion. Moreover, it is a known cofactor for the enzymes involved in glucose metabolism and antioxidant reactions [22]. Additionally, we recognized the influence of iron, which leads to insulin deficiency and induces insulin resistance, as well as causes hepatic dysfunction [23].

## 2. Trace Elements and Type 2 Diabetes Mellitus

### 2.1. Zinc (Zn)

Zinc is an essential element and the second most abundant trace metal in the human body. It appears in all enzyme classes and is a crucial cofactor for many enzymes. Zinc plays a critical role in the synthesis, storage, secretion, and conformational integrity of insulin [24]. Low zinc levels might affect the production and secretion of insulin by pancreatic islet cells. Higher urinary zinc levels indicated increased urinary zinc excretion and lower plasma zinc levels, affecting the progression of diabetes [25,26]. Increased urinary excretion and decreased intestinal absorption of said element cause diabetic hypozincemia, with decreased zinc tissue storage. Some of the recent studies stated that zinc supplementation might have a hypoglycemic effect, modulating other metabolic disorders and antioxidant reactions. Moreover, supplemental zinc reportedly lowers the risk of cardiovascular complications of the disease and helps restore gastrointestinal homeostasis [27,28,29].

Researchers [30] proposed a theory that links increased zinc levels and insulin resistance. It involves a disturbance in hormonal homeostasis through β-cell functioning changes and the modulation of insulin secretion. Another hypothesis concludes that lower zinc levels lead to an upregulation of its transporters, and this may further promote increased zinc uptake [31]. Scientists reported that there might be a correlation between high zinc levels and the prevalence of previously undiagnosed type 2 diabetes. The newest theory focuses on the polymorphism in zinc transporter SLC30A8/ZnT8 and the likely increased incidence of type 2 diabetes. The findings indicate that zinc homeostasis is important for health management and metabolic disease prevention. Moreover, in the conducted studies, zinc transporter ZIP13 showed the ability to regulate beige adipocyte tissue synthesis, further indicating zinc’s crucial role in obesity and metabolic syndrome treatment [32]. Contrary to these hypotheses, other studies reported a decrease in blood and urinary zinc levels among patients with type 2 diabetes [33].

In the performed analysis, scientists selected 16 out of 12,136 publications regarding zinc intake and its role in type 2 diabetes mellitus incidence, using stratified meta-analyses and meta-regressions to assess the sources of heterogeneity and the covariates. As a summary, they concluded that there is no association between zinc dietary consumption and supplementation and T2DM. However, a moderately high dietary zinc intake was able to reduce the overall risk of T2DM by 13% and by up to 41% in rural areas. On the other hand, high serum zinc concentrations were linked to a 64% increased risk of T2DM [34,35]. A systematic review of published studies reporting mechanisms of action of zinc in diabetes was published with the Preferred Reporting Items for Systematic Reviews and Meta-Analyses (PRISMA). Scientists used the following terms to search in the article title, abstract, or keywords: (“Zinc” or “Zn”) and (“mechanism” or “mechanism of action” or “action” or “effect” or “pathogenesis” or “pathology” or “physiology” or “metabolism”) and (“diabetes” or “prediabetes” or “sugar” or “glucose” or “insulin”. They concluded that zinc has antioxidant properties, and its supplementation enhances the activity and levels of antioxidant enzymes and proteins, while significantly reducing lipid peroxidation, and it also plays an important role in the normal functioning of the islet cells of the pancreas. β-cells and their granules are extremely rich in zinc. This further indicates the need for randomized double-blinded placebo-controlled clinical trials conducted for an adequate duration to establish zinc’s therapeutic efficacy and safety in humans [36]. The contradictory findings might therefore result from the element’s levels.

### 2.2. Copper (Cu)

This trace element is heavily involved in antioxidative reactions, and it is a component of copper/zinc superoxide dismutase (Cu/Zn SOD), enabling cellular free radical clearance. Contrary to these beliefs, increasing evidence points out the prooxidant role of Cu. Scientists have stated that high Cu levels in rodents with diabetes caused oxidative stress reactions and renal dysfunction. Despite the increase in Cu/Zn SOD activity, there is a persistence in lipid peroxidation which suggests that Cu does not protect diabetic patients against reactive oxygen species [37,38]. Both copper overload and deficiency can lead to adverse health outcomes. The effect of copper on oxidation can vary, depending on the level of intake. Said metal can act as an antioxidant or a prooxidant. The disruption in Cu homeostasis leads to the impaired metabolism of Zn and as a result, increases oxidative stress reactions in diabetes [39]. Copper can provide a stable active site, further leading to oxidative stress reaction aggravation and playing a role in the development of diabetes, as copper toxicity might induce insulin resistance by acting as a prooxidant [40,41]. Scientists reported that concentrations of copper in the scalp and blood were higher in patients with diabetes than in those without said disease [40]. A different comparative analysis, in which subjects were subdivided into obese and non-obese groups, and diabetic subjects were also subdivided into controlled and uncontrolled groups, hypothesizing that there is a possibility of a positive association between plasma copper levels and diabetic incidence [42]. Furthermore, researchers revealed in yet another study that serum copper was indeed related to the occurrence of the said type of diabetes, but on the contrary, the same could not be said about its urinary levels [43]. Newer research detected positive and coherent associations between the levels of copper in urine, diabetes incidence, hyperglycemia, and elevated fasting plasma glucose levels [44]. Different studies investigated the possible associations between the levels of total blood mercury and methylmercury and diabetes occurrence in correlation with higher selenium intake. Exposure–response analyses, which were assessed with logistic regression and restricted cubic splines, showed an initial decrease in the odds of diabetes, but ultimately, the results showed the inverse connection between total blood mercury and methylmercury levels and diabetic adults, with the associations being modified by selenium. Additionally, it was indicated that higher ingestion of omega-3 fatty acids was linked to a lower incidence of diabetes in patients with previous exposure to mercury [45].

### 2.3. Iron (Fe)

Iron is an abundant metal, making up about 5% of Earth’s crust. The human body consists of about 4.5 g of iron, mainly in the form of hemoglobin, but also in various enzymes controlling intracellular oxidation processes [46]. There are several possible mechanisms of iron involvement in diabetic pathogenesis, such as insulin deficiency, insulin resistance, and dysfunction of the liver. Iron can also become a prooxidant molecule, and through catalyzation of hydroxyl radical formation, it can further lead to the destruction of cell membranes, proteins, and DNA, contributing to the pathogenesis of diabetes [47]. Ferroptosis is a form of regulated cell death which concludes the accumulation of iron-dependent lipid peroxide and disruption in a redox reaction, with a disturbance in its enzyme functioning, particularly that of glutathione peroxidase 4. Ferroptosis occurs in both physiologic and pathogenic reactions. It may lead to the impairment of glucose-stimulated insulin secretion and arsenic-induced pancreatic damage, which then influences diabetic pathogenesis. Moreover, iron and its sulfur react, resulting in further iron deposits in the mitochondria. Said accumulation leads to the production of reactive oxygen species, endoplasmic reticulum stress, disrupted insulin synthesis, and eventually, ferroptosis in the pancreatic β-cells. Additionally, ferroptosis is said to cause diabetic complications such as cardiomyopathy and myocardial ischemia [48].

In the conducted study, iron showed a strong connection with the prevalence of new-onset type 2 diabetes, which, after corrections and further testing, was reported to be of little significance [26]. Other reports stated that patients with diabetes exhibited higher iron concentrations in their hair, blood, plasma, and erythrocytes, but similarly, the variations were not substantial [41,49]. Yet, it is worth mentioning that ferritin and transferrin are better markers of iron status, and an increase in ferritin levels has been reportedly linked to the increased risk of type 2 diabetes [50]. A different study observed a connection between ferritin levels and diabetes, but such an association was not found for serum iron [51]. Another study, which included participants from a general Danish population, measured iron biomarkers such as total plasma iron, ferritin, transferrin saturation, and transferrin. The obtained data showed no consistent correlation between any of the iron biomarkers and diabetes. It is, however, worth mentioning that the markers could have been influenced by coexisting inflammation [52]. Yet another study investigated both serum and urinary chromium and iron levels in Northeast Chinese individuals with impaired fasting glucose levels, impaired glucose tolerance, and type 1 and type 2 diabetes. Scientists found no significant differences in iron and chromium levels among the mentioned groups [53]. Another population-based study with multivariable linear regression models was built to assess the associations of ferritin and transferrin saturation with blood levels of glucagon-like peptide-1, insulin, homeostatic model assessment-insulin resistance, fasting plasma glucose, and hemoglobin. This study stated that the markers of insulin resistance are strongly related to the markers of iron metabolism in healthy subjects, and that these relationships were inconsistent and weaker for short-term and long-term glucose levels. The study further underlined oxidative stress, induced by increased iron deposition in beta- and liver cells, as a possible mechanism leading to cell damage and liver-mediated insulin resistance, higher insulin secretion, and glucose dysregulation [54].

### 2.4. Selenium (Se)

Selenium is an important trace element contained in selenoproteins, which further take part in the formation of antioxidant enzymes such as glutathione peroxidase and thioredoxin reductases. It is worth mentioning that a lower selenium intake and its deficiency are considered to be a risk factor for various cancers, cardiovascular diseases, diabetes, nephropathy, and etc. [55]. We cannot identify a linear relationship between the incidence of type 2 diabetes mellitus and selenium concentrations. Said association is best described as both dose-dependent and chemical form-dependent, according to a U-graph. Too low, as well as too high, selenium intakes may lead to increased diabetes risk. In order to avoid over-supplementation, the baseline levels of Se should be measured [56]. Molecular mechanism studies in animals indicated that both selenoprotein excess and deficiency may potentially play a great role in the pathogenesis of insulin resistance and the dysfunction of β-cells. However, there are some limitations in their translational significance. Selenium dietary intake and supplementation are not likely to be a major risk factor for diabetes development in humans. Nevertheless, its high supranutritional doses should be included as a rather minor contribution combined with other risk factors. Elevated selenium levels in diabetic patients are more likely a consequence of the disease [57]. The association between serum and plasma selenium and the risk of diabetes was described in several studies, such as the one conducted by Rayman and Stranges [58]. On the contrary, other authors did not report any correlation between serum selenium and diabetes [59]. Another study stated that the incidence of diabetes was negatively associated with high toenail selenium levels [60]. Different results showed the connection between urinary selenium and elevated fasting plasma glucose levels but did not report the same association with the risk of diabetes and hyperglycemia [44]. It is worth mentioning that among individuals with a higher intake of selenium, scientists indicated an inverse association between diabetes, total blood mercury, and blood methylmercury, and the interactions of selenium with diabetes were reportedly significant [45].

### 2.5. Magnesium (Mg)

Mg^2+^ is one of the most common intracellular ions, as well as one of the most abundant elements on Earth. Mild to moderate hypomagnesemia has been associated with hypertension, metabolic syndrome, and type 2 diabetes mellitus [61]. Magnesium is a calcium antagonist which can protect vascular endothelial cells against oxidative stress. Imbalances in its levels lead to the inhibition of glucose transporter type 4 translocation, an increase in insulin resistance, and the disruption of lipid metabolism. Thus, hypomagnesemia leads to the initiation and progression of diabetes and the development of its macrovascular and microvascular complications [62].

Scientists reported that a diet with the right supplementation of Mg^2+^ can, through the decrease in blood pressure, hyperglycemia, and hypertriglyceridemia, improve metabolic syndrome. This is possible thanks to gene expression and protein synthesis modulation. Moreover, magnesium is said to have a positive influence on the intestinal microbiota and the metabolism of crucial vitamins such as B1 and D [63]. In conclusion, the protective role of magnesium may consist of a decrease in adipose tissue accumulation, improvement of both glucose and insulin metabolism, and the reduction of inflammatory processes [64]. The aim of the next study was to assess the ability of Mg supplementation to improve glycemic control in patients with T2D. After the dietary stabilization phase and sex, age, fasting blood sugar, and Mg levels stratification, patients were randomly divided into two groups. The control group did not receive Mg supplements, whereas the intervention group was supplemented with 250 mg/day of elemental Mg for three months. As a result, the daily Mg intake significantly improved the levels of glycemic control indicators when compared with those of the control group; hence, oral supplementation of Mg can possibly reduce insulin resistance [65]. In another study with a similar aim—the investigation of oral Mg supplementation in diabetic patients or patients with higher disease susceptibility—focused on the meta-analysis of several databases, with the results being reported as standardized mean differences. The supplementation reduced fasting plasma glucose in diabetic patients when compared to the results for a placebo. Moreover, in people at high risk of diabetes, Mg supplementation significantly improved plasma glucose levels. To conclude, Mg supplementation not only has a potentially beneficial role, improving glucose parameters in people who already suffer from diabetes, it may also improve insulin-sensitivity parameters in patients with increased diabetic risk [66]. When it comes to urinary magnesium levels, diabetics reportedly have increased urinary magnesium losses, which are especially significant in patients with poor control of said disease and glucosuria [67].

### 2.6. Cadmium (Cd)

Cadmium is a natural element which can be found in small quantities in air, water, soil, and food. Exposure to cadmium is mostly occupational. The major routes of its introduction are inhalation of dust and fumes, cigarette smoking, and food ingestion, and cadmium compounds are associated with an increased lung cancer risk. The excretion of cadmium via the urinary system is a biomarker of prolonged exposure. The well-known toxic effects of cadmium intake are renal dysfunction and bone damage. However, diabetes and hypertension incidence have not been categorically found to be a result of this exposure [68]. An experiment carried out on rodents by Bell et al. observed that exposure to cadmium can lead to an increase in blood glucose levels [69]. Another large cross-sectional study reported that urinary cadmium was significantly and dose-dependently associated with impaired fasting glucose levels and the risk of diabetes [70]. Conversely, a study performed in Thailand found that urinary cadmium levels may be connected to hypertension but not to diabetic incidence [71].

Scientists reported that there is a positive correlation between the increased levels of cadmium and the prevalence of previously undiagnosed type 2 diabetes. This finding is in line with the results of previous studies which observed higher urinal cadmium levels among patients with a known diabetes diagnosis [72]. On the other hand, a Swedish study that researched cadmium levels in blood did not find any significant link to the prevalence of type 2 diabetes [73]. It is worth mentioning that Swedish scientists reported slightly lower levels of cadmium (medians 0.24 and 0.27 μg/L for men and women, respectively, in their cohort) when compared to those from another study (median 0.35 μg/L in controls), with both levels being below the average blood concentration for not smoking Europeans, ranging from 0.5 to 1.0 μg/L [74]. Likewise, the South Korean report, despite geometric mean cadmium blood levels in patients with diabetes and high cadmium concentrations in the controls, did not find any significant link between said concentrations and diabetic incidence [26,75].

### 2.7. Bromine (Br)

Bromine is a rare chemical element of the Earth’s crust, although because of the high solubility of its ion, it also accumulates in the oceans. Large amounts of bromide salts are toxic and can lead to a condition called bromism. Nonetheless, bromine is also an essential trace element which has a crucial role in collagen synthesis and development [76]. Studies have explored a connection between higher levels of silver, as well as lower levels of bromine, and the incidence of newly-diagnosed type 2 diabetes. After the corrections, the involvement of bromine appeared to be insignificant [26]. Recently, another study investigated the role of alternative flame retardants such as novel brominated flame retardants (NBFRs) and organophosphate flame retardants (OPFRs). Said retardants are common in the environment and may cause endocrinal disorders. The research was a case-control type study, with 344 participants aged 25–80 years from East China. Researchers assessed the potential connections between serum NBFR and OPFR levels and diabetes mellitus type 2. After some adjustments, serum concentrations of pentabromotoluene, 2,3-dibromopropyl 2,4,6-tribromophenyl ether, tri-n-propyl phosphate, triphenyl phosphate, and tris (2-ethylhexyl) phosphate showed a positive association with diabetic incidence. The control group showed that decabromodiphenyl ethane and triphenyl phosphate were positively linked with fasting plasma glucose levels, as well as triglycerides and high-density lipoprotein cholesterol concentrations. Moreover, a positive significant relationship was found between the flame retardant mixtures and high-density lipoprotein cholesterol levels, with ecabromodiphenyl ethane playing the biggest role in the overall effect. To conclude, exposure to NBFRs and OPFRs may lead to the promotion of type 2 diabetes mellitus occurrence [77]. In yet another study, with multivariate logistic regression models adjusted for potential confounders, among the participants with a BMI ≥ 25 kg m^−2^, urinary Br-THM levels were significantly (*p* < 0.001) higher in diabetics than in healthy individuals [78].

### 2.8. Barium (Ba)

This alkaline earth metal is used in metallurgy, and its compounds are heavily involved in the production of petroleum and radiology. Barium compounds, which can dissolve, are toxic to humans through their interference with potassium ion channel functioning. Our knowledge about the effects of barium exposure is mainly based on animal studies. Human data is sparse, especially for prolonged and lower dosage exposures. Scientists have indicated that cardiovascular and kidney diseases, as well as metabolic and neurological disorders, are post-exposure effects of barium intake [79].

A study was carried out to determine whether occupational exposure to barium, among other metals, was related to type 2 diabetes mellitus. The research was based on a group of coke oven workers who were exposed to high concentrations of polycyclic aromatic hydrocarbons (PAHs) and heavy metals. The results showed that urinary barium levels were associated with the occurrence of hyperglycemia [44]. As the mitochondrial DNA copy number (mtDNA-CN) is believed to be related to cell metal exposure and metabolism, researchers conducted a study to verify the role of mtDNA-CN, together with plasma levels of selected metals, in diabetes risk exacerbation. The study group consisted of the elderly population, and a 6-year follow-up study was performed. Metal concentration levels were obtained through inductively coupled plasma mass spectrometry (ICP-MS), and mtDNA- CN was measured by real-time PCR. With the use of regression models, low plasma levels of both Ba and mtDNA-CN were associated with T2DM incidence in the elderly. Moreover, Ba concentrations were positively linked to mtDNA-CN. The study highlights the importance of further examination of the mtDNA-CN, metal levels, and T2DM incidence [80].

### 2.9. Lead (Pb)

This relatively unreactive element displays a weak metallic character that is illustrated by its amphoteric nature. Lead is extensively used in construction, batteries, bullets and shots, leaded gasoline, and radiation shielding. Said element is a neurotoxin that can accumulate in soft tissues and bones. It damages the nervous system and disrupts biological enzyme functioning, which may then lead to various neurological disorders, as well as cardiovascular and renal system problems. Lead, through the disruption of glucose uptake, is connected to higher fasting blood sugar levels, metabolic syndrome occurrence, and increased diabetes risk [81]. Scientists reported that elevated levels of urine Pb were positively associated with an increased diabetic risk [82]. A study determined that Pb can promote diabetic development in obese rodent models. It was observed that after 8 weeks, said metal exposure contributed to the occurrence of fasting hyperglycemia. After 12 weeks, the rodents developed glucose intolerance. Moreover, a possible direct effect of the metal on hepatic gluconeogenic genes was discovered, as the exposed animals showed elevated expression of the PEPCK and glucose-6-phosphatase genes [83]. Another study aimed to establish the possible relationship between blood Pb level and possible risk factors, such as body mass index insulin resistance and diet, with fasting blood sugar levels in women living in the Indonesian mining area. The study involved women aged 30–49, selected through previously performed sampling. Collected data showed that blood Pb concentration could not be associated with fasting blood sugar levels [84].

A different study assessed the effects of concurrent exposure to lead and cadmium on the risks of diabetes and kidney function impairment. The exposure levels for both metals were established as low to moderate, valued through mean concentrations of Cd and Pb ([Cd]b = 0.59 µg/ and [Pb]b = 4.67 µg/dL). A total of 71 out of 176 study subjects presented with high fasting plasma glucose levels. The diagnosis of diabetes was 4.2 times higher in subjects with a higher exposure profile and 2.9-fold higher in patients with the established glomerular filtration ≤ 60 mL/min/1.73 m^2^. Moreover, the prevalence odds ratio for albuminuria was five times higher in subjects with plasma glucose levels above 180 mg/dL. In conclusion, the obtained results suggest that exposure to both Cd and Pb impacts kidney function and increases the risk of diabetes [85]. Regarding occupational exposition to lead, urinary concentrations might play a substitutional role. In this regard, research reported that levels of urinary lead were higher in patients with diabetes than in healthy individuals. Moreover, scientists observed that urinary lead has a significant association with the risk of hyperglycemia [44,86].

### 2.10. Arsenic (As)

Arsenic is best classified as nonmental, while some of its elemental forms are metal-like. The toxicity of said chemical element varies from the exceptionally poisonous arsine to elemental arsenic itself, which is relatively inert. Arsenical compounds can cause skin irritation, dermatitis, carcinogenesis, and neurological diseases. The most common poisoning comes from oral intake. Recently, more and more studies have indicated arsenic involvement in type 2 diabetes mellitus induction. Arsenic-induced diabetes is said to be caused by inflammation, oxidative stress, and apoptosis. Scientists highlight dysfunction of the pancreatic β-cells, impaired insulin secretion, insulin resistance, and the reduction of cellular glucose transport as possible association mechanisms [87,88]. A study performed in Bangladesh reported that arsenic exposure elevates fasting blood glucose levels and insulin resistance, and subsequently, the risk of hyperglycemia, with the arsenic exposure-related reduction of skeletal muscle mass being the underlying mechanism. Moreover, females are reportedly more susceptible to these effects than are males [89]. The National Institute of Environmental Health Sciences stated that there is a possible association between inorganic arsenic, high drinking water arsenic concentrations, and type 2 diabetes mellitus occurrence [90]. Another study, with a diametrically different approach, found no association between said element and the disease, but it is worth noting that the levels of arsenic in drinking water in the area of conducted research were much lower than the levels where the connection has been reported [26,91]. Previous research, which determined arsenic exposure through its urinary levels, presented contrary outcomes, some of which significantly associated them with the heightened incidence of type 2 diabetes [92], while others did not find any substantial validity to this hypothesis [93]. The results of a more recent study revealed that urinary arsenic may be positively associated with elevated fasting plasma glucose (FPG) levels but not with the risk of diabetes and hyperglycemia, although the authors suggest that this result might be obtained due to the younger age of the research group and its smaller size [44]. Studies performed on rodents examined the role of in-utero arsenic exposure with diabetes in the offspring. Scientists observed elevated post-natal fasting glucose in exposed fetuses, as well as elevated insulin resistance and microRNA and DNA methylation profiles in the pathways related to diabetes. Animal studies stated that adequate B vitamin supplementation may lower the disease risk induced by arsenic [94].

### 2.11. Strontium (Sr)

Researchers have linked the development of type 2 diabetes mellitus (T2DM) to strontium’s role in antioxidation and adipose metabolism [95]. Strontium plays a crucial role in bone metabolism, but its impact on glucose and lipid metabolism remains largely unexplored. Chen et al. [96] included a total of 1448 newly diagnosed T2DM patients, 782 patients with impaired glucose regulation (IGR), and 2230 matched controls with normal glucose tolerance in their study. Patients with T2DM and IGR showed significantly lower plasma strontium concentrations compared to those of the control group. The median concentrations were 35.8 mg/L, 37.9 mg/L, and 40.8 mg/L, respectively (*p* < 0.001). As strontium concentration increased, the odds of T2DM and IGR decreased significantly. A plateau followed this decrease. In addition, plasma strontium showed a negative correlation with total cholesterol, low-density lipoprotein cholesterol, and lipid peroxidation. The human body primarily stores strontium in the bones. Strontium readily moves between blood and bone in humans, with its concentration in bone closely linked to levels in the bloodstream [97]. Plasma strontium could potentially serve as a biomarker for strontium homeostasis.

Prior research on multiple-metal exposure has primarily focused on the association between strontium and T2DM, yet the findings have been inconsistent [26,98,99]. Researchers in China discovered that patients with type 2 diabetes (n = 122) exhibited higher plasma strontium levels than those of the control group (n = 429) [98]. Furthermore, Norwegian research revealed no discernible variation in blood strontium levels between individuals with T2DM and those without the condition [99]. Animal studies indicate that strontium may play a role in inhibiting adipogenesis. It likely occurs because of the activation of peroxisome proliferator-activated receptors in adipocytes. The disorders of lipid metabolism, inflammation, and oxidative stress play a crucial role in the development of insulin resistance and diabetes [100,101]. After a year of treatment, a study discovered that strontium ranelate had no impact on the fasting plasma glucose concentrations of 40 postmenopausal women with osteoporosis. Furthermore, it had no impact on hemostasis factors or lipid profiles [102]. Environmental and lifestyle factors, such as the quality of drinking water and smoking, may also play a role in the elevated Sr levels observed in individuals with type 2 diabetes [103]. The prevalence of type 2 diabetes showed a significant inverse association with urinary Sr levels (OR = 0.39; 95% CI = 0.23 to 0.64) [104]. Research has shown that Sr may have beneficial effects in managing diabetes by promoting antioxidant activity and regulating fat metabolism [105,106]. In diabetic mice, Sr can regulate the expression of pancreas- and kidney-related genes and enhance insulin tolerance by controlling glucose levels [100].

### 2.12. Nickel (Ni)

Ni exposure is commonly found at low levels in the general population. The concentration in blood under normal environmental exposure typically ranges from 0.001 to 1.29 mmol/L in the general population of industrialized nations [107]. In 2022, a study found no significant link between Ni levels and glucose in individuals with T2DM. According to the study, there was no notable distinction in serum concentrations of Ni between the patients with T2DM and the control subjects (4.4 vs. 4.4 g/L, *p* < 0.05) [108]. A recent study conducted in Norway, known as HUNT3, discovered a significant correlation between nickel levels and both T2DM and undiagnosed cases of T2DM. The researchers advised caution in interpreting the results due to the potential for syringes to leak nickel ions into the samples [99]. Several published studies indicate that individuals with diabetes tend to have lower levels of nickel in their bloodstreams compared to those without the condition [109,110]. Studies on rats and humans have demonstrated that a diet deficient in nickel can negatively impact growth, reproductive capabilities, and blood glucose levels. Additionally, it can also lead to changes in the distribution of other essential elements in the body, such as calcium, iron, and zinc [109]. A 2014 study in China found that individuals with T2DM had higher levels of nickel in their urine than those without T2DM. The median nickel concentration was found to be 4.03 mg/L in T2D subjects, whereas it was 3.40 mg/L in non-T2D subjects (*p* < 0.01). Researchers found a link between higher urinary nickel levels and elevated levels of fasting plasma glucose, insulin, HbA1C, and HOMA-IR [111]. Previous studies have shown that prolonged oral exposure to nickel leads to significant metal accumulation in the kidneys. The order of nickel accumulation in various organs, from highest to lowest, is the kidneys, lungs, liver, and heart. Research findings indicate a positive relationship between blood nickel and urine nickel levels (r = 0.3) [103]. On the other hand, blood nickel levels may primarily indicate recent exposure as compared to urinary nickel levels, as nickel has a short half-life in this system [112]. It is important to note that serum nickel levels primarily indicate exposure to soluble nickel compounds, rather than insoluble nickel salts or unabsorbed metallic nickel deposits found in the lung [113]. A study conducted in Pakistan found that individuals with diabetes, both male and female, had higher levels of Ni in their scalp hair. Significant differences were observed based on age. There were no significant differences between the mean values of Ni in the blood. However, the levels were slightly higher for older men (1.7–3.1 μg/L) and women (1.2–3.56 μg/L) compared to the corresponding controls (1.3–2.5 and 1.11–1.99 μg/L) [41].

Various tissues release free radicals when exposed to Ni [114]. In rats, Ni causes a significant increase in the production of nitric oxide synthase (iNOS) from the pancreas, resulting in hyperglycemia. Additional research on animal models has demonstrated that Ni can elevate blood sugar levels through various mechanisms, such as increased hepatic glycogenolysis, heightened pancreatic glucagon release, gluconeogenesis, or reduced peripheral glucose consumption [115]. Numerous studies have established a link between circulating Ni and a higher risk of T2DM [111,114]. In 2017, researchers made an initial report on the discovery of high concentrations of Ni in islets or other human tissue. The pancreatic insulin-producing islets of Langerhans have a significant concentration of nickel (median concentration 132.1; IQR 42.5 to 232.5 nmol/g protein) [115,116].

### 2.13. Aluminum (Al)

Al (III) tends to accumulate in the tissues of individuals with diabetes and appears to speed up the formation of β-sheets when measured over extended incubation periods [116,117,118].

Researchers have established a connection between the onset of type II diabetes and the irregular fibrillation of human islet amyloid polypeptide (hIAPP). The formation of oligomers and fibrils by hIAPP, a 37-amino acid residue polypeptide, has widely recognized detrimental effects on pancreatic islet cells [119,120]. Aluminum ions have the ability to induce rapid precipitation of the amyloid peptide and modify the morphology of the resulting aggregates [121]. The serum aluminum concentration in diabetic patients was found to be 22.8 ± 18.1 μg/L, which is significantly higher compared to the concentration of 11.4 ± 5.1 μg/L observed in healthy subjects [122]. Moreover, research has indicated that aluminum impacts the formation rate and structure of proislet amyloid polypeptide, the precursor peptide of hIAPP [123]. The interaction between Al(III) and hIAPP at His18-Al(III)-His18 sites can promote the nucleation and fibrillation processes. EGCG can hinder this process by forming amorphous aggregates with hIAPP through π–π stacking with aromatic residues. By interacting with Phe23 residues and adding a positive charge, the Al(III)/EGCG complex can effectively stop hIAPP fibrillation. This interaction leads to electrostatic repulsion between adjacent hIAPP monomers, resulting in a more efficient blocking effect compared to that of EGCG alone. Additionally, the Al(III)/EGCG complex has the potential to transform toxic fibrils into harmless amorphous aggregates. These findings could be a valuable reference for developing new inhibitors of toxic hIAPP fibrils for diabetes management [124]. An in vitro study found that incubating aluminum at 37 °C for 120 h led to a 19.3% faster formation of the beta-pleated sheet structure in hIAPP [125]. When compared to the control group, Serdar et al. discovered higher plasma Al levels in patients with impaired glucose tolerance, impaired fasting glucose, and type 2 diabetes mellitus. Furthermore, there was a clear link between plasma Al levels, plasma glucose, and HbA1c levels [126]. Levine et al. found a frequent occurrence of Al toxicity in individuals with impaired renal function, as well as a higher accumulation of Al in diabetic patients’ tissues. Diabetes mellitus linked high levels of Al to decreased activity of calmodulin in the heart muscle, potentially leading to reduced functions of the sarcoplasmic reticulum (Ca + Mg)-ATPase and calcium transport. Aluminum toxicity enhances the negative effects of diabetes by reducing sarcoplasmic reticulum calcium uptake [127]. In a Chinese study, after adjustment for potential confounders, urinary aluminum was associated with altered FPG, IFG, or diabetes risk (*p* < 0.05). Furthermore, after additional adjustments for multiple testing, aluminum was no longer significantly associated with the outcomes [128]

### 2.14. Chromium (Cr)

An important micronutrient for blood sugar management and insulin activity, chromium is needed for both of these processes. It functions as an essential antioxidant that preserves insulin homeostasis. Antioxidants including chromium are depleted, and free radical generation is elevated in type 2 diabetes mellitus (T2DM). Previous research has suggested a link between poorer glycemic management and low serum levels of chromium [129]. While the daily consumption of chromium in Western countries may be adequate, foods from Asia may contain insufficient amounts of chromium or compounds that prevent it from being absorbed, which could explain why Asians exhibit lower serum chromium concentrations [130]. Significant numbers of individuals worldwide display low serum levels of chromium without having a clinically apparent chromium deficiency [131]. Rajendran K. et al. presented a study that indicated that regardless of HbA1c readings, patients with type 2 diabetes mellitus showed lower serum chromium levels. When comparing uncontrolled type 2 diabetes to controlled type 2 diabetes, the amount of chromium in the former was significantly lower, at 0.103 ± 0.04 in healthy patients vs. 0.065 ± 0.03 in T2DM patients [132]. Hajra B. et al. study included 200 subjects, 100 T2DM patients and 100 nondiabetics. The healthy individuals displayed higher serum levels of chromium than did the diabetic patients [133]. Exercise, diet, and stress are some of the other factors that impact plasma chromium levels. Assessing the impact of these confounding factors on the chromium levels in diabetic patients was not feasible [134].

### 2.15. Manganese (Mn)

Involved in several biological functions, such as energy metabolism; antioxidant function; detoxification; musculoskeletal, immunological, and reproductive systems; bone growth; and the control of brain and nerve function, manganese is a “structurally” important trace mineral [135]. Mn affects gluconeogenesis and insulin release, according to the results of rat studies [136]. Both the excess and lack of manganese impair the metabolism of carbohydrates. When there is dietary stress, an increase in Mn consumption helps because it minimizes endothelial dysfunction, lowers oxidative stress, and enhances insulin secretion [137]. The levels of the homeostasis model assessment (HOMA) and Mn consumption were found to be significantly positively correlated. According to the study’s authors, supplying children with enough Mn can help them avoid developing insulin resistance and type 2 diabetes in the future. A few studies demonstrate the connection between low Mn levels and impaired glucose metabolism in the elderly [138,139]. There is contradictory research regarding the connection between Mn and diabetes. According to several investigations, there is no statistically significant correlation between diabetes and magnesium levels [49,140]. It is worth mentioning that manganese status might be different within general populations according to some previously conducted studies, and therefore, its association with diabetes might change The link between Mn and diabetes has been examined in the recently released study from 2022 [127]. A population of 2575 hypertensive people who were recruited from China participated in the study. The risk of diabetes was studied by the authors. In the population under study, diabetes prevalence seems to be 27.0%. Furthermore, in the group with hypertension, a U-shaped relationship between serum manganese and diabetes was noted, with sex considerably altering the correlation. Plasma Mn and T2DM appear to have a U-shaped connection, meaning that T2DM is more likely to occur at both high and low Mn levels [141,142]. In a 5-year Japan Collaborative Cohort Study, scientists reported that there is a strong inverse association between dietary manganese intake and the risk of type 2 diabetes in women but not men, and the association was observed mainly for those with low iron intake, particularly premenopausal women [143,144].

### 2.16. Calcium (Ca)

The most prevalent mineral in the human body is calcium. The majority of the calcium in the body is stored in the skeleton, but calcium that is free and hydrated in solution plays a crucial role as a physiological mediator in a variety of metabolic and regulatory processes. In clinical medicine, the free cation concentration in the extracellular fluid—formerly known as ionized calcium—is routinely measured in patients who may or may not have known disorders involving calcium metabolism [145]. Diabetes affects calcium homeostasis, which leads to issues with cell control in skeletal, cardiac, platelet, and erythrocyte muscles. The compromised homeostasis is alarming since it may play a major role in regulating appropriate insulin secretion and action, which in turn may influence different vascular problems on its own [146,147]. Changes in calcium and vitamin D levels seem to play a role in the development of type 2 diabetes (T2DM), as demonstrated by Pittas et al. in 2007 [148]. There are not many cohort studies examining high serum calcium levels as indicators of poor glucose metabolism. One such study showed that people with higher serum calcium concentrations had a higher risk of developing diabetes. The study found that throughout follow-up, serum calcium levels generally increased in 77 instances of type 2 diabetes. These findings are consistent with the results of earlier cross-sectional studies that found higher serum calcium levels in diabetic patients compared to non-diabetic individuals. These differences persisted, even after excluding those who took calcium supplements or had calcium levels outside of the normal range, indicating a significant correlation between elevated serum calcium levels and an increased risk of type 2 diabetes [149]. In 1329 middle-aged and elderly Korean patients, a different investigation verified the prevalence of diabetes and metabolic syndrome with higher serum calcium levels (*p* < 0.001). Age, sex, body mass index (BMI), serum creatinine, phosphorus, parathyroid hormone (PTH), 25-OHD, smoking, alcohol consumption, exercise, total energy, and calcium and sodium intake did not influence this connection [150]. The findings of Zhai Z et al. imply a causal relationship between increased serum calcium levels and an increased risk of type 2 diabetes [151]. Hypercalciuria is a clinical presentation of early diabetes and can be reduced with insulin therapy. Urinary calcium excretion is decreased in diabetic patients with renal impairment, and urinary calcium excretion cannot possibly predict renal dysfunction progression in patients with type 2 diabetes mellitus [152].

## 3. Conclusions

**Figure 1:**
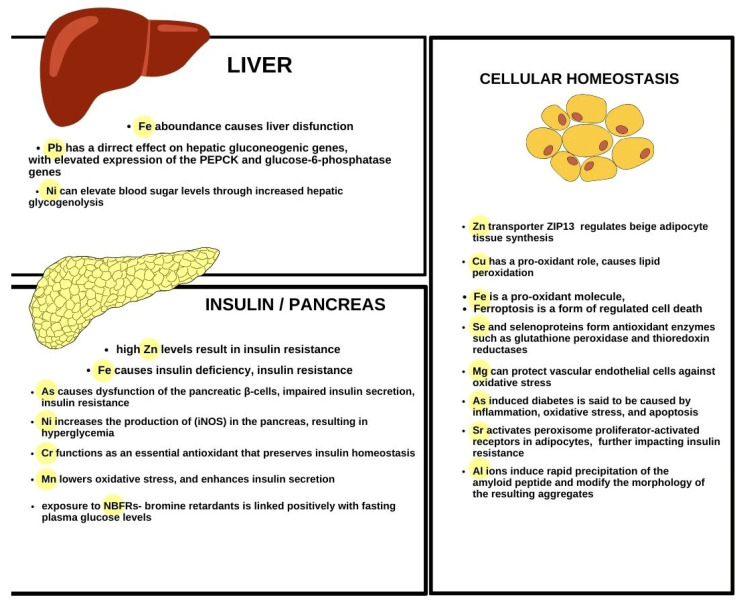
The description of known associations between the pathogenesis of metabolic disturbance in type 2 diabetes mellitus and selected metals, with the inclusion of the most affected organs and metabolic values.

Metals can affect metabolic pathways and therefore, are a force to be reckoned with when it comes to disease pathogenesis and potential treatment strategies. Constant waste and untreated chemical effluent contamination lead to a rise in their emissions. Cadmium, aluminum, arsenic, barium, copper, lead, nickel, and iron are linked to a wide range of health hazards. Heavy metals often cause reactive oxygen species development and inhibit antioxidant defense mechanisms. They accumulate in the liver, kidney, and pancreas, deteriorating glucose metabolism, glycolysis, glycogenesis, and gluconeogenesis by impairing enzymatic activity, and hepatic glucose homeostasis damage is a major risk of diabetes mellitus occurrence. Furthermore, impaired liver, pancreas, and kidney functions lead to elevated blood glucose levels. Calcium homeostasis not only regulates insulin secretion and action but is also affected in the course of T2DM. When it comes to promising future treatment strategies development, Mn supplementation could possibly aid in avoiding insulin resistance and diabetes development. Sr, on the other hand, may have beneficial effects in managing diabetes by promoting antioxidant activity and regulating fat metabolism Moreover, scientists reported lower chromium concentrations in diabetic patients. The associations between blood serum/plasma levels and urine concentrations of the aforementioned trace elements are listed in Table 1 and Figure 1 and the concentration ranges (μg/L) of trace elements in whole blood, serum, and urine are presented in Table 2 (Table 1 and Table 2) [153]. The associations between metal concentrations and diabetes cannot imply causation without the extension of further research. More studies including human participants may further lead to a better understanding of the disease pathogenesis and the development of promising treatments, as well as prevention methods.

## Figures and Tables

**Table 1:** A summary of known associations between type 2 diabetes mellitus and metals.

Element	Blood Plasma/Serum Levels	Study Characteristics	Urine Concentrations	Study Characteristics
Zinc	Both excessively low and high levels may be associated with diabetes development. Correct supplementation may have beneficial outcomes [25,30].	Population-based studies	Diabetics show a significant hyperzincuria as compared with levels of the controls [34].	Population-based study
Copper	Higher levels are positively associated with diabetes [39,40].	Population-based studies	Higher levels are positively associated with diabetes, hyperglycemia, and fasting plasma glucose [44].	Population-based study
Iron	Increased ferritin levels are linked to the increased risk of type 2 diabetes [51,54].	Population-based studies	No significant differences in iron levels were noted among participants [53].	Population-based study
Selenium	Both selenoprotein excess and deficiency may potentially play a great role in the pathogenesis of insulin resistance and the dysfunction of β-cells [57].	Animal study	A positive association was noted between higher levels and elevated fasting plasma glucose levels, but not with the risk of diabetes and hyperglycemia [44].	Population-based study
Magnesium	Hypomagnesemia leads to the initiation and progression of diabetes and the development of its vascular complications [62]. Oral supplementation of Mg can possibly reduce insulin resistance [65,66].	Population-based studies	Lower urine concentrations were noted in patients with poor control of said disease and glucosuria [67].	Population-based study
Lead	Higher levels are positively associated with diabetes [83].	Animal study	Higer levels are positively associated with hyperglycemia and diabetes risk [82,86].	Population-based studies
	Higher levels are positively associated with diabetes [85].	Population-based study		
Cadmium	Higher levels are positively associated with diabetes [72]. No association was found in these studies [73,75].	Population-based study	Higer urinary levels are positively associated with diabetes [72].	Population-based study
Arsenic	Higher levels are linked to fasting blood glucose level elevation and insulin resistance [89].	Population-based study	Higher levels may be positively associated with elevated fasting plasma glucose levels but not with the risk of diabetes and hyperglycemia [44].	Population-based study
Bromine	Exposure to NBFRs and OPFRs is positively associated with T2DM risk [77].	Population-based study	Urinary Br-THM levels were higher in diabetics than in healthy individuals [78].	Population-based study
Barium	Higher levels are positively associated with diabetes [80].	Population-based study	Higher urinary levels were positively associated with hyperglycemia [44].	Population-based study
Strontium	Patients with T2DM and IGR showed significantly lower plasma strontium concentrations compared to those of the control group [97].	Population-based study	The prevalence of type 2 diabetes showed a significant inverse association with urinary Sr levels [105,106].	Population-based studies
Nickel	Higher levels are positively associated with diabetes [99].		Higher levels are positively associated with diabetes [109].	Population-based study
	No significant association was noted [108].	Population-based studies		
	There is a link between circulating Ni and a higher risk of T2DM [115].	Animal study		
Aluminum	Higher plasma levels were noted in patients with impaired glucose tolerance, impaired fasting glucose, and type 2 diabetes mellitus [122].	Population-based study	No significant association was noted [128].	Population-based study
Chromium	Lower levels of chromium are found in diabetic patients [132,133].	Population-based study	No significant differences in chromium levels were noted among participants [53].	Population-based study
Manganese	Supplementation can possibly cease the development of insulin resistance and type 2 diabetes. Plasma Mn and T2DM appear to have a U-shaped connection, meaning that T2DM is more likely to occur at both high and low Mn levels [138,141,143,144].	Population-based study	Higher urinary Mn levels were found to be associated with higher fasting blood glucose [141].	Population-based study
	No association has been found [140].	Population-based study		
Calcium	Higher levels are positively associated with diabetes [149,151].	Population-based study	Hypercalciuria is a clinical presentation of early diabetes [152].	Population-based study

**Table 2:** Concentration ranges (μg/L) of trace elements in whole blood, serum, and urine based on the study in Ref. [153]. We included the data from the 5th and 95th percentiles.

Element	Whole Blood	Serum	Urine Concentrations
Aluminum	<2–2.8	0.53–1.23	<2–6.6
Arsenic	0.07–3.4	0.03–1.73	1.1–44
Barium	0.15–1.2	0.24–1.95	0.3–11
Bromine	1252–2847	1650–3660	511–4430
Cadmium	0.13–1.7	<0.009–0.017	<0.1–0.58
Calcium	48,000–59,600	88,000–102,000	21,800–227,000
Chromium	<0.036–0.09	<0.03–0.08	<0.1–0.35
Copper	729–1360	795–1949	1.7–15
Iron	395,000–514,000	-	<1–18
Lead	5.4–26.3	0.01–0.1	0.095–1.0
Magnesium	30,200–39,000	18,600–23,500	5010–130,300
Manganese	5.0–13.5	0.39–0.6	<0.06–0.12
Nickel	<0.35–0.6	0.19–0.4	<0.4–4.0
Selenium	85–128	70–109	3.3–30
Strontium	9.4–41	18–73	30–286
Zinc	4730–6730	733–1110	25–685
